# Association of cholecystectomy with osteoporosis risk: a prospective study using data from the UK Biobank

**DOI:** 10.3389/fendo.2023.1259475

**Published:** 2023-10-20

**Authors:** Qin Yang, Ming Wang, Tongtong Zhang, Jun Wen, Lu Long, Congying Xia

**Affiliations:** ^1^Section for HepatoPancreatoBiliary Surgery, Department of General Surgery, The Third People’s Hospital of Chengdu and The Affiliated Hospital of Southwest Jiaotong University, Chengdu, China; ^2^Department of General Surgery, The Third People’s Hospital of Chengdu and The Affiliated Hospital of Southwest Jiaotong University, Chengdu, China; ^3^Department of Epidemiology and Health Statistics, West China School of Public Health and West China Fourth Hospital, Sichuan University, Chengdu, Sichuan, China; ^4^Department of Cardiology, West China Hospital, Sichuan University, Chengdu, China

**Keywords:** cholecystectomy, osteoporosis, cohort, risk factor, gender difference, interaction

## Abstract

**Objective:**

To investigate whether prior cholecystectomy is associated with incident osteoporosis.

**Background:**

Cholecystectomy may have consequences involving abnormal metabolism. Studies investigating the association between prior cholecystectomy and osteoporosis have yielded inconsistent results.

**Methods:**

In total, 17,603 UK Biobank participants underwent cholecystectomy, and 35,206 matched controls were included in this study. They were followed up for incident osteoporosis, which was determined using ICD-10 codes (M80–82). The association between cholecystectomy and osteoporosis was assessed using Cox proportional regression modeling. The association between osteoporosis risk and cholecystectomy was further analyzed across age, sex, serum vitamin D level, and body mass index (BMI) categories.

**Results:**

Within a median follow-up period of 13.56 years, 3,217 participants were diagnosed with osteoporosis. After adjustment for relevant confounders, prior cholecystectomy was associated with a 1.21 times higher risk of osteoporosis in women (hazard ratio (HR): 1.21 [95% CI, 1.12–1.31], p < 0.001) and a 1.45 times higher risk in men (HR: 1.45 [95% CI, 1.10–1.90], p = 0.007). In women, the association was stronger for patients who were aged 40–55 years, with BMI < 18.5 kg/m^2^, and vitamin D between 30 and 50 nmol/ml. No significant interactions between cholecystectomy and income level, education level, presence of hypertension, or diabetes were identified in either sex.

**Conclusions:**

Our findings indicated that people who underwent cholecystectomy had a higher risk of developing osteoporosis after adjustment for potential confounders. Our findings suggest that awareness of the risk of osteoporosis in patients with a history of cholecystectomy is merited.

## Introduction

Cholecystectomy, one of the most common abdominal surgical procedures, is widely performed in patients with chronic symptomatic gallstones and/or cholecystitis (1, 2). Currently, some researchers advise expanding indications of early prophylactic cholecystectomy to selected asymptomatic gallstone patients (3). Although laparoscopic cholecystectomy is known to be safe, awareness of perioperative and relatively long-term complications is important in establishing surgical indications.

Prior studies have found that cholecystectomy may cause abnormal metabolism, including disturbed glucose levels and diminished absorption of fat-soluble vitamins, especially vitamin D (4–7). Vitamin D deficiency is found to be associated with an increased risk of osteoporosis and consequently with vulnerability to fractures, which can cause very severe health burdens, including death (8–11). Nevertheless, to date, the relationship between cholecystectomy and osteoporosis is still controversial.

A recent nationwide registered study found that the risk of osteoporosis was not increased after cholecystectomy (12). Similarly, a prior study revealed that bone mineral density, a marker for osteoporosis, did not differ between cholecystectomy and control groups even though lower vitamin D levels were observed after cholecystectomy (5). In contrast, Lee et al. reported that prior cholecystectomy was associated with an increased risk of fractures in the Korean population (13).

Therefore, we designed this case–control study to prospectively investigate the association between prior cholecystectomy and the risk of incidental osteoporosis using data from the UK Biobank.

## Methods

### Study design and population

**Figure 1:**
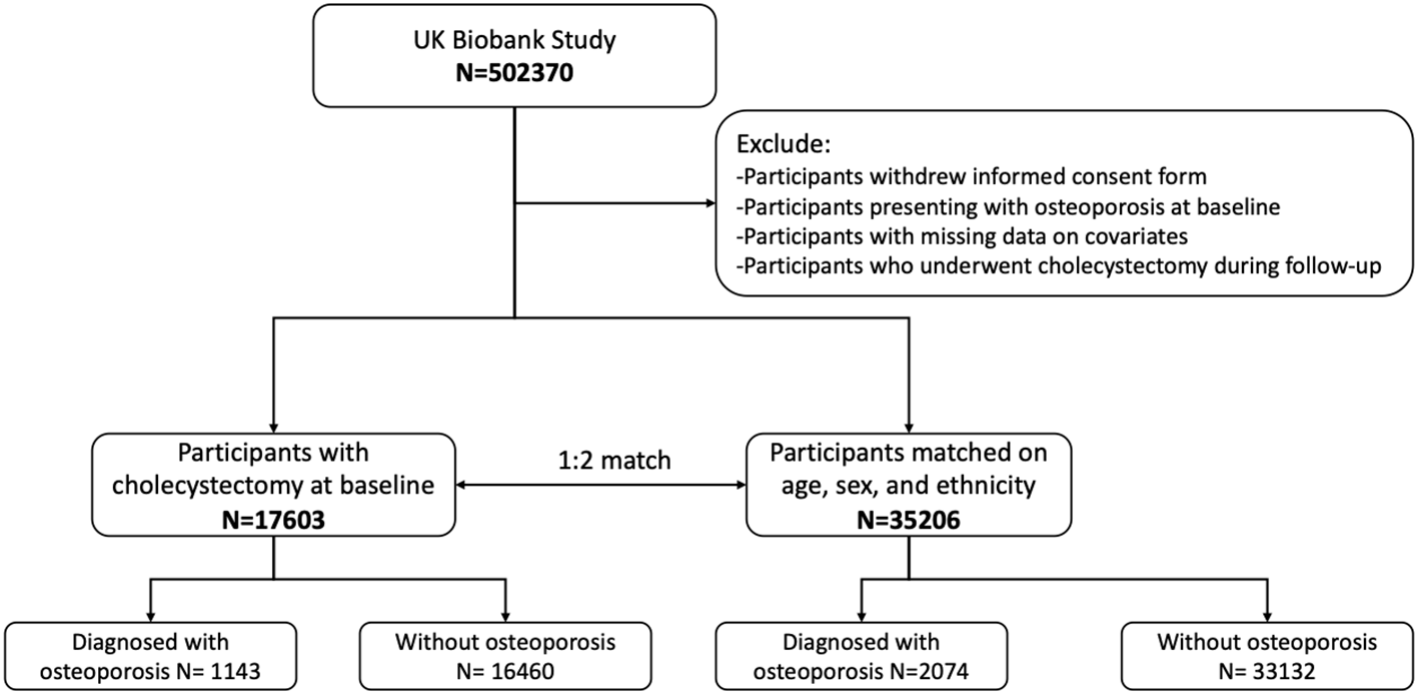
Flowchart illustrating the selection of the study sample.

This study was conducted in the framework of the UK Biobank, which is a population-based prospective cohort study that recruited more than 500,000 participants aged 40–69 years in 2006–2010. Detailed information on the study design of the UK Biobank has been published previously (14). In the current study, participants who underwent cholecystectomy at baseline assessment were selected as cases. Having undergone cholecystectomy was defined as a self-reported history of cholecystectomy and/or presentation with the operation procedure codes J18.1–18.3. These cases were then matched on age, sex, and ethnicity to participants from the same database who had not undergone cholecystectomy, in a ratio of 1:2. Participants who underwent cholecystectomy during the follow-up period and those who presented with osteoporosis prior to the baseline assessment were excluded. We further excluded participants who withdrew from the UK Biobank study and those with missing data on the covariates. Overall, 17,603 cases and 35,206 controls were included in this study and were followed up continuously until the date of their first incidence of osteoporosis or until October 31, 2022. The follow-up period lasted up to 15.54 years. A flowchart of the study cohort is presented in **Figure 1**.

The current study was conducted under UK Biobank application number 93810. The UK Biobank received ethical approval from the NHS National Research Ethics Service North West (11/NW/0382; 16/NW/0274), the National Information Governance Board for Health and Social Care in England and Wales, and the Community Health Index Advisory Group in Scotland. In addition, an independent Ethics and Governance Council was formed in 2004 to oversee the UK Biobank’s continuous adherence to the Ethics and Governance Framework that was developed for the study (http://www.ukbiobank.ac.uk/ethics/). All participants provided written informed consent.

### Outcomes

Participants were monitored continuously for the occurrence of osteoporosis, which was defined using the International Classification of Disease, 10th revision (ICD-10) codes M80 (osteoporosis with pathological fracture), M81 (osteoporosis without pathological fracture), and M82 (osteoporosis in diseases classified elsewhere). ICD codes were retrieved from hospital inpatient records, which contain information on inpatient episodes of care for England, Wales, and Scotland (for detailed information: https://biobank.ndph.ox.ac.uk/showcase/).

### Covariates and rationale for selection

Covariates were selected based on prior publications or known confounders of the association between cholecystectomy and osteoporosis. Information on ethnicity, education level, income level, smoking status, and alcohol consumption were obtained using touch-screen questionnaires. Body mass index (BMI) was calculated as weight (in kilograms) divided by the square of height (in meters) and participants were classified as underweight (<18.5 kg/m^2^), normal weight (18.5 to 24.9 kg/m^2^), overweight (25 to 29.9 kg/m^2^), or obese (≥30 kg/m^2^). Vitamin D levels were assessed based on 25-hydroxyvitamin D (25(OH)D) concentration in serum. Diabetes mellitus (DM) was defined based on insulin or oral hypoglycemic agent use and/or a fasting plasma glucose level ≥7.0 mmol/L. Hypertension was defined as systolic pressure ≥140 mmHg and/or diastolic pressure ≥90 mmHg, and/or current use of antihypertensive medication. Further details on these measurements can be found in the UK Biobank online protocol (http://www.ukbiobank.ac.uk).

### Statistical analyses

Baseline characteristics are reported in the form of descriptive statistics. Data are presented in the form of means with standard deviations (SDs) or median and interquartile range for continuous variables (depending on the distribution), and in the form of numbers and percentages for categorical variables. Differences between groups were compared using unpaired t-tests or the chi-squared test when appropriate. Follow-up time was defined as the period between the time of enrollment and the date of first diagnosis of osteoporosis, date of loss to follow-up, mortality date, or October 1, 2022, whichever occurred first. A total of 132 participants who were lost to follow-up and 5,194 participants who died before developing osteoporosis were censored in the analyses. Associations between cholecystectomy and incident osteoporosis were assessed using Cox proportional hazards models, and the results are reported in the form of hazard ratios (HRs) with their 95% confidence intervals (95% CIs). Three incremental models that included different covariates were fitted: model 1 was unadjusted; model 2 included age, sex (only for the whole cohort), and ethnicity; and model 3 additionally adjusted for smoking status, income level, education level, BMI, alcohol intake frequency, hypertension, diabetes mellitus, serum cholesterol, and vitamin D level.

Subgroup analyses were conducted to examine the associations between cholecystectomy and incident osteoporosis among pre-specified subgroups based on the following: sex; age category (40–55, 55–65, or >65 years); BMI category (<18.5, 18.5–24.9, 25–29.9, or ≥30 kg/m^2^); smoking status (never, previous, current, or unknown); income level (<£18,000, £18,000–30,999, £31,000–51,999, £52,000–100,000, or >£100,000); alcohol intake frequency (never, monthly or less, two to four times per month, or two to three times per week); vitamin D level (<30, 30–50, or >50 nmol/L); hypertension (presence or absence); and diabetes (presence or absence). Fully adjusted Cox proportional hazards models were built for each subgroup.

Data were analyzed using R (Version 4.3, R Foundation, Vienna, Austria). Statistical significance was defined as a p-value <0.05.

## Results

The baseline characteristics of participants by sex and presence of cholecystectomy are shown in **Table 1**. Overall, compared with controls, patients who had undergone cholecystectomy were more likely to report never having smoked, and had higher BMI, a higher prevalence of diabetes mellitus, lower cholesterol levels, and lower vitamin D levels (**Table 1**).

**Table 1:** Baseline characteristics by cholecystectomy.

Baseline characteristics	Overall	Control	Cholecystectomy	p-Value
	N = 52,809	N = 35,206	N = 17,603	
Age (years), mean (SD)	59.56 (7.13)	59.56 (7.13)	59.56 (7.13)	>0.999
Sex (male, %)	11,937 (23%)	7,958 (23%)	3,979 (23%)	>0.999
Ethnicity, n (%)				>0.999
White	51,291 (97%)	34,194 (97%)	17,097 (97%)	
Black	141 (0.3%)	94 (0.3%)	47 (0.3%)	
Asian	618 (1.2%)	412 (1.2%)	206 (1.2%)	
Other	549 (1.0%)	366 (1.0%)	183 (1.0%)	
Unknown	210 (0.4%)	140 (0.4%)	70 (0.4%)	
Income level				<0.001
< 18,000	12,371 (23%)	7,923 (23%)	4,448 (25%)	
18,000 to 30,999	12,249 (23%)	8,195 (23%)	4,054 (23%)	
31,000 to 30,999	10,300 (20%)	7,007 (20%)	3,293 (19%)	
52,000 to 100,000	6,700 (13%)	4,654 (13%)	2,046 (12%)	
> 100,000	1,643 (3.1%)	1,210 (3.4%)	433 (2.5%)	
Unknown	9,546 (18%)	6,217 (18%)	3,329 (19%)	
Education level				<0.001
College or university degree	14,664 (28%)	10,547 (30%)	4,117 (23%)	
A levels/AS levels or equivalent	5,349 (10%)	3,623 (10%)	1,726 (9.8%)	
O levels/GCSEs or equivalent	11,852 (22%)	7,845 (22%)	4,007 (23%)	
CSEs or equivalent	2,311 (4.4%)	1,462 (4.2%)	849 (4.8%)	
NVQ or HND or HNC or equivalent	3,157 (6.0%)	2,016 (5.7%)	1,141 (6.5%)	
Other professional qualifications	3,345 (6.3%)	2,173 (6.2%)	1,172 (6.7%)	
Unknown	12,131 (23%)	7,540 (21%)	4,591 (26%)	
Smoking status, n (%)				<0.001
Never	28,799 (55%)	19,567 (56%)	9,232 (52%)	
Previous	19,005 (36%)	12,431 (35%)	6,574 (37%)	
Current	4,712 (8.9%)	3,025 (8.6%)	1,687 (9.6%)	
Unknown	293 (0.6%)	183 (0.5%)	110 (0.6%)	
Alcohol intake frequency, n (%)				<0.001
Never	1,777 (3.4%)	1,051 (3.0%)	726 (4.1%)	
Monthly or less	2,661 (5.0%)	1,659 (4.7%)	1,002 (5.7%)	
2–4 times per month	3,068 (5.8%)	2,108 (6.0%)	960 (5.5%)	
2–3 times per week	4,356 (8.2%)	3,181 (9.0%)	1,175 (6.7%)	
4 or more times per week	4,238 (8.0%)	3,186 (9.0%)	1,052 (6.0%)	
Unknown	36,709 (70%)	24,021 (68%)	12,688 (72%)	
BMI (kg/m^2^), mean (SD)	28.01 (5.25)	27.13 (4.79)	29.77 (5.65)	<0.001
Hypertension, n (%)	24,560 (47%)	16,250 (46%)	8,310 (47%)	0.022
Diabetes mellitus, n (%)	4,189 (7.9%)	2,211 (6.3%)	1,978 (11%)	<0.001
Cholesterol (mmol/L), mean (SD)	5.65 (1.03)	5.71 (1.02)	5.52 (1.06)	<0.001
Vitamin D (nmol/L, mean (SD)	48.90 (20.88)	49.79 (20.97)	47.10 (20.57)	<0.001

Categorical variables are presented as number (percentage). Continuous variables are presented as mean (standard deviation).

BMI, body mass index; AS, Advanced Subsidiary; GCSE, General Certificate of Secondary Education; NVQ, National Vocational Qualification; HND, Higher National Diploma; HNC, Higher National Certificate.

**Figure 2:**
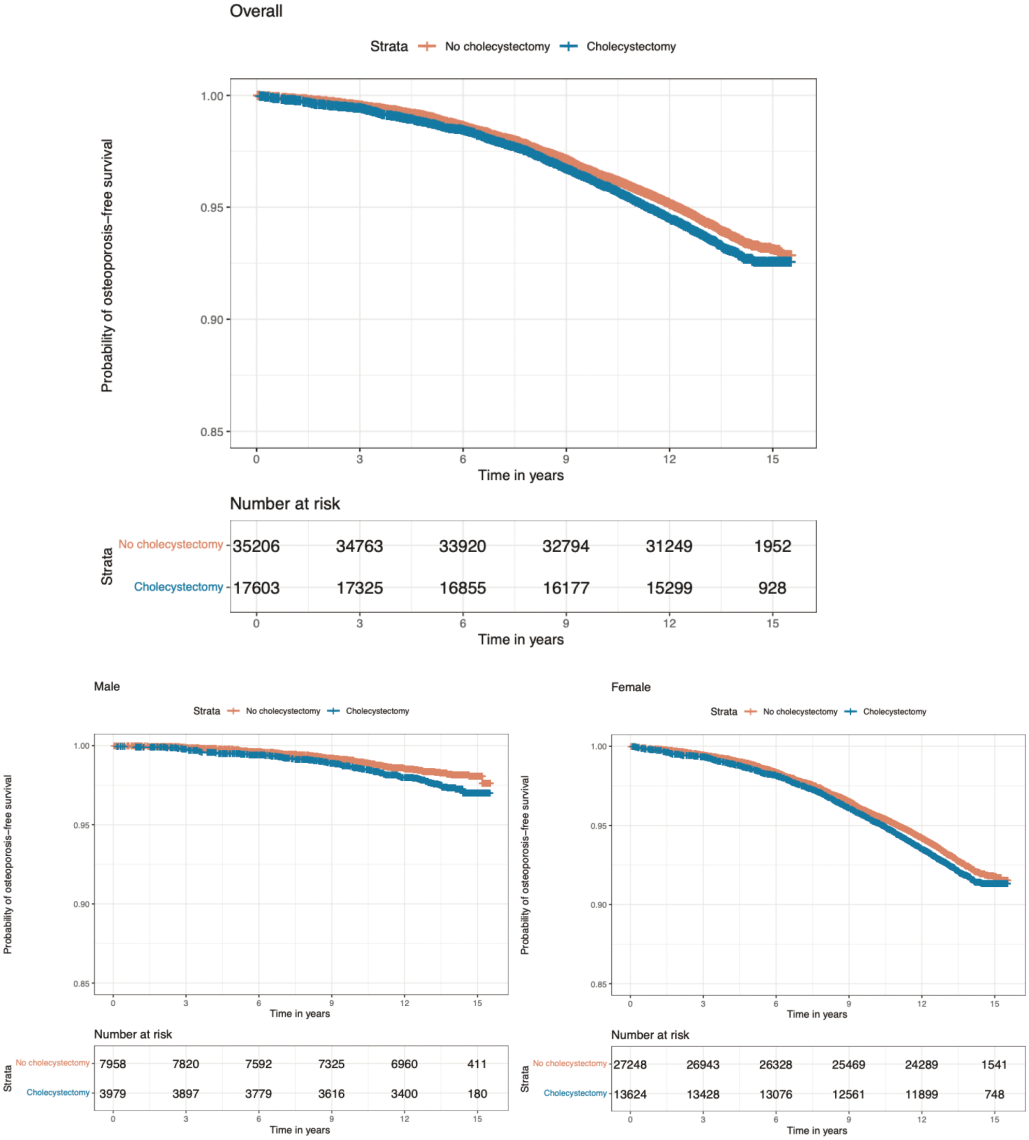
Probability of survival free of osteoporosis according to presence of cholecystectomy. p = 0.004 for the difference between cholecystectomy and no cholecystectomy in terms of incidence of osteoporosis in overall population. p = 0.005 for the difference between cholecystectomy and no cholecystectomy in terms of incidence of osteoporosis in men. p = 0.03 for the difference between cholecystectomy and no cholecystectomy in terms of incidence of osteoporosis in women.

During a median follow-up period of 13.56 years (interquartile range: 12.69 to 14.32 years), 1,143 participants (6.5%) in the cholecystectomy group and 2,074 participants (5.9%) in the control group developed osteoporosis. The cumulative incidence of osteoporosis was significantly higher in participants who had undergone cholecystectomy than in those without cholecystectomy (log-rank test, p = 0.004) (**Figure 2**). Further adjustment for potential confounders, including age, sex, ethnicity, smoking status, income level, education level, BMI, alcohol intake frequency, hypertension, diabetes mellitus, total cholesterol levels, and vitamin D level, did not substantially attenuate the association (**Table 2**). Compared with participants who had not undergone cholecystectomy at baseline, the multivariate-adjusted HR for osteoporosis after cholecystectomy was 1.23 (95% CI, 1.14–1.32) in the whole cohort, 1.21 (95% CI, 1.12–1.31) for women, and 1.45 (95% CI, 1.10–1.90) for men (**Table 2**). The association between cholecystectomy and osteoporosis was stronger in men than in women (p < 0.001 for interaction with sex).

**Table 2:** Hazard ratios with 95% CI for the association between cholecystectomy and incident osteoporosis.

	Total			Women			Men		
	Control	Cholecystectomy	p-Value	Control	Cholecystectomy	p-Value	Control	Cholecystectomy	p-Value
Model 1	Ref	1.11 (1.04–1.20)	0.004	Ref	1.09 (1.01–1.17)	0.026	Ref	1.46 (1.12–1.91)	0.005
Model 2	Ref	1.11 (1.03–1.20)	0.004	Ref	1.09 (1.01–1.17)	0.026	Ref	1.46 (1.12–1.91)	0.005
Model 3	Ref	1.23 (1.14–1.32)	<0.001	Ref	1.21 (1.12–1.31)	<0.001	Ref	1.45 (1.10–1.90)	0.007

Model 1 was unadjusted. Model 2 was adjusted for age, sex (for the whole cohort only), and ethnicity. Model 3 was additionally adjusted for smoking status, income level, education level, body mass index (BMI), alcohol intake frequency, hypertension, diabetes mellitus, serum total cholesterol, and vitamin D level.

**Figure 3:**
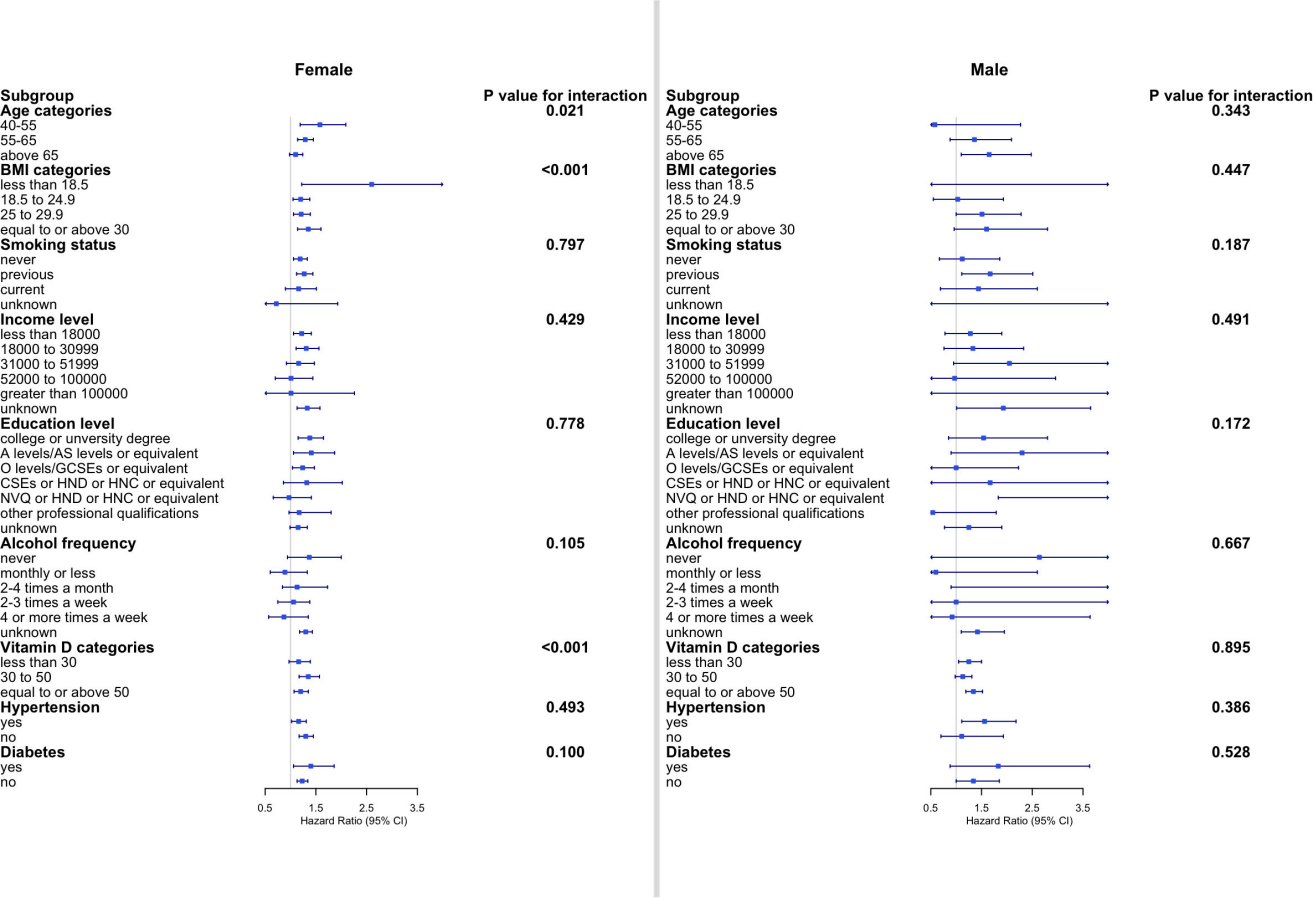
Subgroup analyses of the association between cholecystectomy and osteoporosis according to potential baseline risk factors. Fully adjusted Cox proportional hazards models were built including age, ethnicity, smoking status, income level, education level, alcohol intake frequency, hypertension, diabetes mellitus, serum cholesterol, and vitamin D level for each subgroup. CI, confidence interval; BMI, body mass index.

Subgroup analyses were subsequently performed according to sex and other baseline risk factors (**Figure 3**). In women, statistically significant differences across strata were observed for age, BMI, and serum vitamin D level (all p-values <0.05 for interaction), with a stronger association observed in women who were aged 40–55 years, with BMI <18.5 kg/m^2^, and with vitamin D between 30 and 50 nmol/ml. In men, however, the associations were generally similar across subgroups stratified according to age category, BMI category, smoking status, income level, education level, alcohol consumption, vitamin D level, and presence of hypertension and diabetes mellitus (all p-values for interaction >0.05, **Figure 3**).

## Discussion

This study prospectively examined the association between cholecystectomy and osteoporosis in female and male participants from the UK Biobank. Our findings showed that of the incidence of osteoporosis was higher in participants who underwent cholecystectomy than in age- and sex-matched controls.

Several prior studies have investigated the association between prior cholecystectomy and the risk of osteoporosis and/or fractures, yielding inconsistent results. In a study of the Korean population from 2010 to 2015, cholecystectomy was associated with a higher risk of fractures considered to be a clinical consequence of osteoporosis (13). Another study reported that prior cholecystectomy was associated with osteoporosis as reflected by lower bone mineral density based on X-ray images in the selected population, with a limited sample size (6). Our findings in a large European population favor a positive association between prior cholecystectomy and risk of osteoporosis, which is consistent with these studies. In contrast, another study in the Danish population failed to find an association between prior cholecystectomy and the risk of fractures (12). This discrepancy may be explained by differences between the study populations and differences in the determination of outcomes. We used a strict definition of osteoporosis, excluding fracture-related ICD-10 codes, while the Danish study only used ICD codes for fractures. Given the fact that ICD-10 codes alone cannot distinguish osteoporotic fractures from fractures attributable to other causes, our assessment of the association between prior cholecystectomy and osteoporosis may be more precise.

We found that prior cholecystectomy was associated with an increased risk of incident osteoporosis independent of serum vitamin D levels. Several previous studies have found that cholecystectomy is associated with vitamin D deficiency. Researchers have therefore speculated that cholecystectomy disturbs the absorption of vitamin D and this may lead to osteoporosis (5, 6, 15–17). We also observed significantly lower levels of serum vitamin D in both men and women after cholecystectomy. However, in our study, participants who had undergone cholecystectomy still had a higher risk of incident osteoporosis than controls after adjustment for vitamin D level. Another potential mechanism may be that changes in the gut microbiota following cholecystectomy affect bone metabolism, possibly through the immune system (18, 19). Further studies are warranted for a better understanding of the potential underlying etiological link between cholecystectomy and increased risk of osteoporosis.

In addition, we found that the association between prior cholecystectomy and incident osteoporosis was stronger in men. This may be related to sex hormones and sex-specific differences in bone biology and morphology. Despite the fact that osteoporosis mainly affects women, men may present with more risk factors for secondary osteoporosis (20). In women, we observed an increased risk of incident osteoporosis after cholecystectomy in younger age groups, for unknown reasons. In theory, the elderly may be more predisposed to other comorbidities as compared to younger individuals. Therefore, the effect of cholecystectomy may not be strong enough to significantly change the risk of osteoporosis as compared with other factors, such as postmenopausal status. We also found that, in women, the association between cholecystectomy and osteoporosis was at its strongest in the lowest BMI category (<18.5 kg/m^2^). Lower-BMI populations have muscle weakness, and insufficient protective padding around the hips could be one reason for increased osteoporotic fractures (12, 21, 22). Additionally, we found that the association between cholecystectomy and osteoporosis was stronger in women who preferred not to report their alcohol consumption. Light-to-moderate alcohol consumption is generally reported to be beneficial for bone health (23, 24). From a clinical point of view, interventions targeting appropriate consumption of alcohol may have potential benefits in offsetting osteoporosis post-cholecystectomy for women. From a biological perspective, further studies are needed to understand the mechanisms by which alcohol consumption influences osteoporosis.

### Strengths and limitations

Our study prospectively assessed the association between prior cholecystectomy and incident osteoporosis in a large number of participants, adjusting for a comprehensive set of potential confounding factors.

The limitations of our study also need to be elucidated. First, we defined osteoporosis strictly using ICD codes with or without fractures, which may be more precise than prior studies that have used a wider definition that included fractures that may not related to osteoporosis. Nonetheless, the use of ICD codes alone may still be imprecise and may produce a biased estimation of incidence rates in cases where little information about patients is available. Second, the UK Biobank has been reported to have selection bias; therefore, our estimates of the incidence of osteoporosis may not be generalizable to the full population of the United Kingdom or to overseas populations. Third, even though our analyses adjusted for a large set of confounders, some of the associations identified might be due to residual or unmeasured confounding, such as the use of vitamin supplements, sun exposure, amount of exercise, and use of hormone replacement therapy among women.

## Conclusion

In summary, prior cholecystectomy is associated with an increased risk of osteoporosis, and this association was found to be independent of serum vitamin D levels. The association is higher in a younger age, smaller BMI group, and lower vitamin D levels for women. Our findings suggest that awareness of the risk of osteoporosis is merited in patients with a history of cholecystectomy. Further studies are needed to elucidate the underlying mechanisms.

## Data availability statement

The UK Biobank is an open access resource and bona fide researchers can apply to use the UK Biobank dataset by registering and applying at http://ukbiobank.ac.uk/register-apply/. Further information is available from the corresponding author upon request. Requests to access the datasets should be directed to yangqin_0813@hotmail.com.

## Ethics statement

The current study was conducted under UK Biobank application number 93810. The UK Biobank received ethical approval from the NHS National Research Ethics Service North West (11/NW/0382; 16/NW/0274), the National Information Governance Board for Health and Social Care in England and Wales, and the Community Health Index Advisory Group in Scotland. In addition, an independent Ethics and Governance Council was formed in 2004 to oversee the UK Biobank’s continuous adherence to the Ethics and Governance Framework that was developed for the study (http://www.ukbiobank.ac.uk/ethics/). All participants provided written informed consent. The studies were conducted in accordance with the local legislation and institutional requirements. The participants provided their written informed consent to participate in this study.

## Author contributions

QY: Conceptualization, Formal analysis, Methodology, Project administration, Resources, Validation, Writing – original draft, Writing – review & editing. MW: Investigation, Validation, Writing – review & editing. TZ: Project administration, Writing – review & editing. JW: Investigation, Writing – review & editing. LL: Conceptualization, Investigation, Methodology, Project administration, Resources, Supervision, Validation, Writing – review & editing. CX: Conceptualization, Data curation, Formal analysis, Methodology, Supervision, Writing – original draft, Writing – review & editing.
